# Establishment of African pygmy mouse induced pluripotent stem cells using defined doxycycline inducible transcription factors

**DOI:** 10.1038/s41598-024-53687-9

**Published:** 2024-02-08

**Authors:** Sumito Matsuya, Kaoru Fujino, Hiroyuki Imai, Ken Takeshi Kusakabe, Wataru Fujii, Kiyoshi Kano

**Affiliations:** 1 Laboratory of Developmental Biology, Joint Graduate School of Veterinary Medicine, Yamaguchi University, Yamaguchi, Japan; 2 Laboratory of Veterinary Anatomy, Joint Faculty of Veterinary Medicine, Kagoshima University, Kagoshima, Kagoshima Japan; 3 Laboratory of Developmental Biology, Joint Faculty of Veterinary Medicine, Yamaguchi University, 1677-1, Yoshida, Yamaguchi Prefecture 7538511 Japan; 4 Laboratory of Veterinary Anatomy, Joint Faculty of Veterinary Medicine, Yamaguchi University, Yamaguchi, Japan; 5 Present Address: Research Institute for Cell Design Medical Science, Yamaguchi University, Yamaguchi, Japan; 6 Laboratory of Biomedical Science, Department of Veterinary Medical Sciences, Graduate School of Agricultural and Life Sciences, The University of Tokyo, 1-1-1 Yayoi, Bunkyo-Ku, Tokyo, 113-8657 Japan; 7 Research Center for Food Safety, Graduate School of Agricultural and Life Sciences, The University of Tokyo, Tokyo, Japan

**Keywords:** Induced pluripotent stem cells, Stem cells, Mammary stem cells

## Abstract

*Mus minutoides* is one of the smallest mammals worldwide; however, the regulatory mechanisms underlying its dwarfism have not been examined. Therefore, we aimed to establish *M. minutoides* induced pluripotent stem cells (iPSCs) using the PiggyBac transposon system for applications in developmental engineering. The established *M. minutoides* iPSCs were found to express pluripotency markers and could differentiate into neurons. Based on in vitro differentiation analysis, *M. minutoides* iPSCs formed embryoid bodies expressing marker genes in all three germ layers. Moreover, according to the in vivo analysis, these cells contributed to the formation of teratoma and development of chimeric mice with *Mus musculus*. Overall, the *M. minutoides* iPSCs generated in this study possess properties that are comparable to or closely resemble those of naïve pluripotent stem cells (PSCs). These findings suggest these iPSCs have potential utility in various analytical applications, including methods for blastocyst completion.

## Introduction

*Mus minutoides*, also known as the African pygmy mouse, has recently gained attention as a domestic pet. *M. minutoides* is considered one of the smallest mammals and belongs to the same genus as the common laboratory mouse (*Mus musculus*). Despite their common biological characteristics, such as lifespan and gestation period, *M. minutoides* is significantly smaller than *M. musculus*, measuring only one-tenth of the body size of *M. musculus*^1^. As previous studies involved the performance of phylogenetic analysis and focused on the sex-determination patterns and viruses carried by *M. minutoides*^2–6^, the dwarfism of *M. minutoides* has not been studied in detail.

In mammals, the Gh–Igf1 axis influences postnatal body size in mammals^7,8^. In our previous study, we analyzed the growth hormones in *M. minutoides*^9^; however, a study exploring the characteristics of growth-related factors, particularly growth hormones, has not been performed. The generation of genetically modified animals, such as knockout mice, is effective for conducting body size regulation-related molecular genetic analyses. However, as *M. minutoides* is not a laboratory animal, its use is difficult, and techniques for developmental engineering have not been established.

Pluripotent stem cells (PSCs) are defined as embryonic stem cells (ESCs) derived from the inner cell mass of blastocysts and induced pluripotent stem cells (iPSCs) reprogrammed from differentiated cells. iPSCs were first produced in *M. musculus* in 2006^10^. *M. minutoides* is vulnerable to transport-related stress. Moreover, as only a few investigators use *M. minutoides*, obtaining individuals is a challenging task. In prior studies with *M. minutoides*, developmental engineering techniques, particularly superovulation, egg collection, and fertilized egg culture systems, were not employed. Consequently, obtaining fertilized eggs from *M. minutoides* to establish ESCs poses a significant challenge. Therefore, this study focused on iPSCs. iPSCs have garnered attention in regenerative medicine and have been applied to developmental process analysis and understanding of pathological conditions^11–13^. The potential applications of iPSCs in conserving endangered wildlife have also been considered in recent years^14,15^. iPSCs have been successfully induced in mice, rats^16^, humans^17^, cattle^18^, pigs^19^, goats^20^, sheep^21^, Bactrian camel^22^, Tokuno-shima spiny rats^23^, American plain voles^24^, and other rodents, including the naked mole rat^25^.

In this study, we aimed to establish *M. minutoides* iPSCs derived from *M. minutoides* fibroblasts. These iPSCs exhibit the requisite attributes of pluripotent stem cells and actively participate in ontogenesis. Overall, *M. minutoides* iPSCs are an important resource that may substantially improve our understanding of dwarfism, which is the main characteristic trait of *M. minutoides*.

## Results

### Establishment of the iPSC lines from fibroblasts

**Figure 1:**
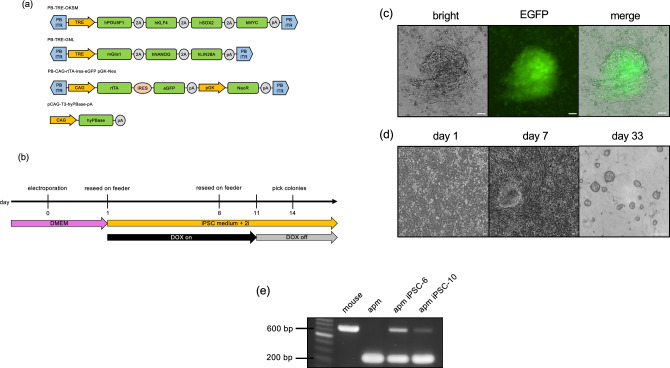
Establishment of *Mus minutoides* induced pluripotent stem cells (iPSCs). (**a**) iPSC plasmid constructs. (**b**) Timeline of the establishment of *M. minutoides* iPSCs. (**c**) Fluorescence microscopic image of *M. minutoides* iPSC-like cells on day 7. Bar: 100 µm. (**d**) Colonies of *M. minutoides* iPSCs on days 0, 7, and 33 after electroporation. Bar: 100 µm. (**e**) PCR identification of *M. minutoides*-derived cells using genomic DNA. "mouse" and "apm” represent genomic DNA extracted from *M. musculus* and *M. minutoides*, respectively.

The four iPSC-inducing plasmid constructs used in this study are shown in Fig. 1a. Electroporation was employed as the plasmid transfer method to establish the *M. minutoides* iPSC strains (Fig. 1b). Primary fibroblasts were established from the tail tip of *M. minutoides*. The day of electroporation was designated as day 0; the *M. minutoides* fibroblasts transfected with the iPSC-inducing plasmid formed colonies and exhibited EGFP^+^ signals on feeder cells by day 7 (Fig. 1c). Upon subsequent passaging, the cells developed naïve ESC-like colonies when cultured with DOX. On day 12, DOX supplementation was discontinued; however, naïve ESC-like colonies were still observed on day 33 after several passages (Fig. 1d). A total of 16 *M. minutoides* iPSC lines were produced, with no apparent differences in growth rate or colony morphology. To confirm the origin of iPSCs from *M. minutoides*, genomic DNA was extracted from a cell line that had undergone at least 10 additional passages, and PCR was performed. *M. minutoides* and *M. musculus* genomes were differentiated based on the disparity in the length of the *Ghr* promoter region (unpublished data). PCR products from the *M. minutoides* genome were detected, confirming that all iPSC lines established in this study originated from *M. minutoides* (Fig. 1e).

### Characteristics of *M. minutoides* iPSCs

**Figure 2:**
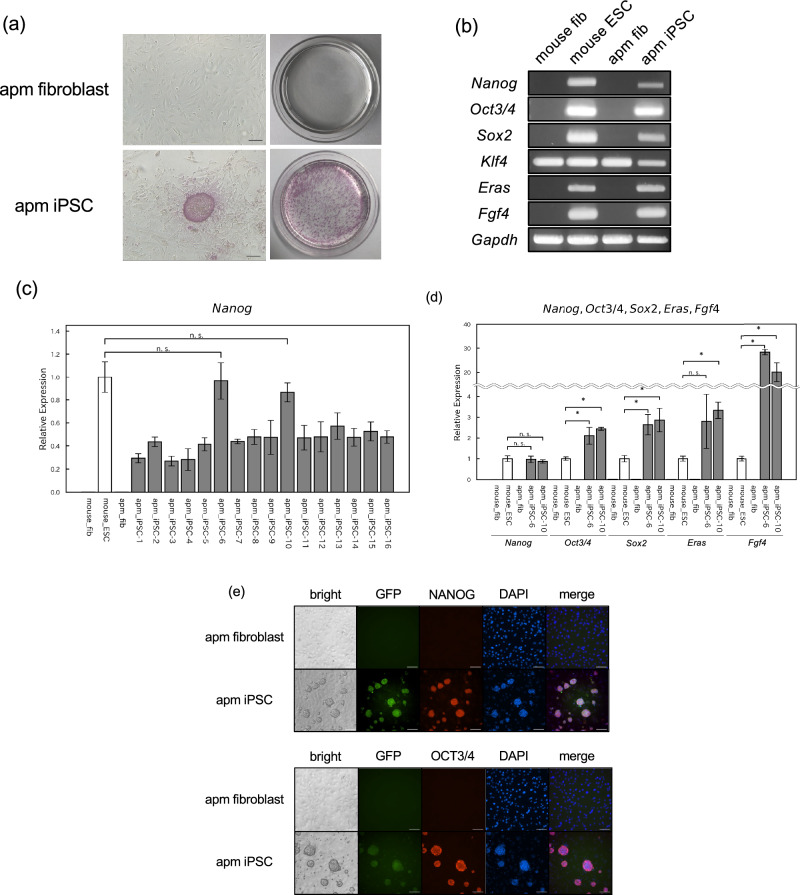
Analysis of pluripotency in *Mus minutoides* induced pluripotent stem cells (iPSCs). (**a**) Microscopic (left) and macroscopic image (right) of alkaline phosphatase-stained *M. minutoides* iPSCs (apm iPSC) in a 60-mm dish. *M. minutoides* fibroblast (apm fibroblast) was used as the negative control. Bar: 100 µm. (**b**) Expression analysis of pluripotency marker genes based on RT-PCR. *Gapdh* was used as the positive control. “mouse” and “apm” represent *M. musculus* and *M. minutoides*, respectively. (**c**) Relative *Nanog* expression in 16 established *M. minutoides* iPSC lines. *Gapdh* was used as the endogenous control, and mouse_ESC expression level was defined as 1.0. “mouse” and “apm” represent *M. musculus* and *M. minutoides*, respectively. (**d**) Relative *Nanog*, *Oct3/4*, *Sox2*, *Eras*, and *Fgf4* expression levels in *M. minutoides* iPSCs (apm_iPSC-6, apm_iPSC-10). *Gapdh* was used as the endogenous control, and mouse_ESC expression level was defined as 1.0. “mouse” and “apm” represent *M. musculus* and *M. minutoides*, respectively. (**e**) Fluorescence immunostaining with the anti-NANOG and -OCT3/4 antibodies in *M. minutoides* iPSCs (apm iPSC). *M. minutoides* fibroblast (apm fibroblast) was used as the negative control. Bar: 100 µm.

To assess the pluripotency of *M. minutoides* iPSCs, we initially examined the activity of ALP, a commonly used stem cell marker. Notably, almost all colonies established in this study were ALP positive (Fig. 2a). cDNA was then synthesized using total RNA extracted from *M. minutoides* iPSCs, and the expression of the pluripotency marker genes was analyzed using RT-PCR. The expression of the pluripotency marker genes, *Eras* and *Fgf4*, was observed in conjunction with the genes delivered by the iPSC-inducing plasmids despite not being introduced through electroporation (Fig. 2b). To confirm the presence of *Fgf4* in *M. minutoides* iPSCs, we performed RT-PCR using cDNA and primers specific to a region of the *Fgf4* gene sequence. Sequencing of the amplified product revealed that several nucleotide sequences differed from those of *M. musculus* (Supplemental Fig. 1a); however, the predicted amino acid sequence derived from the nucleotide sequence was identical to that of *M. musculus* (Supplemental Fig. 1b). Furthermore, we quantitatively evaluated the expression of the pluripotency marker gene, *Nanog*, a representative marker, in multiple established cell lines using RT-qPCR. *Nanog* expression levels varied among the cell lines (Fig. 2c). In addition, we examined the expression of other pluripotency marker genes (*Oct3/4*, *Sox2*, *Eras*, and *Fgf4*) in *M. minutoides* iPSCs derived from lines 6 and 10, in which *Nanog* expression was comparable to that in *M. musculus* ESCs. The expression levels of these markers, particularly *Fgf4*, were significantly higher in *M. minutoides* iPSCs than in *M. musculus* ESCs (Fig. 2d). Correlation analysis of the gene expression levels revealed a weak positive correlation between Eras and other genes (Supplemental Fig. 2). We also examined the expression and localization of the pluripotency markers, NANOG and OCT3/4, using fluorescence immunostaining. Only EGFP-positive *M. minutoides* iPSC colonies stained positive for NANOG and OCT3/4 (Fig. 2e).

**Figure 3:**
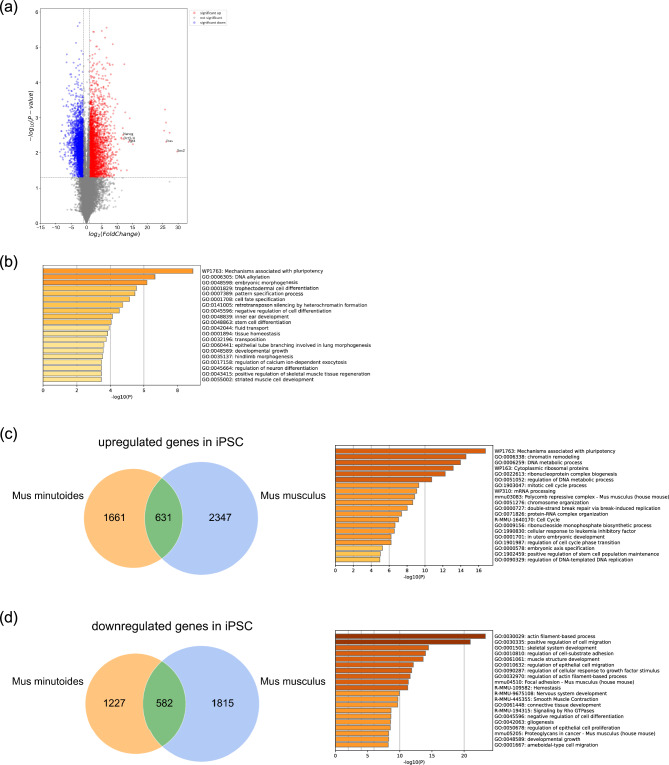
RNA-seq analysis of *M. minutoides* iPS cells and fibroblasts. (**a**) Volcano plot of differentially expressed genes in *M. minutoides* iPSCs and fibroblasts. (**b**) Enrichment analysis of the upregulated genes in *M. minutoides* iPSCs, obtained using Metascape. (**c**) (Left) Venn diagram showing the twofold upregulated genes in *M. minutoides* and *M. musculus* iPSCs compared to fibroblasts. (Right) Enrichment analysis of the upregulated genes in *M. minutoides* and *M. musculus* iPSCs was obtained using Metascape. (**d**) (Left) Venn diagram showing the twofold downregulated genes in *M. minutoides* and *M. musculus* iPSCs compared to fibroblasts. (Right) Enrichment analysis of the downregulated genes in *M. minutoides* and *M. musculus* iPSCs was obtained using Metascape.

To obtain a more in-depth clarification of gene expression in *M. minutoides* iPSCs, RNA-seq analysis was performed using iPSCs and fibroblasts. The 29,437 expressed genes exhibited significant differences in gene expression status between *M. minutoides* iPSCs and fibroblasts. The expression levels of 6503 genes varied significantly by more than twofold, such as *Nanog*, *Oct3/4*, *Sox2*, *Eras*, and *Fgf4*, which were upregulated based on RT-qPCR (Fig. 3a). Enrichment analysis of the differentially expressed genes (DEGs) from *M. minutoides* iPSCs and fibroblasts revealed several Gene Ontology (GO) terms related to pluripotency pathways and cell differentiation (Fig. 3b). Of the 292 genes in WP1763 with the highest rank in the enrichment analysis, 102 were upregulated in iPSC_6 and iPSC_10 compared to the levels found in fibroblasts (Supplemental Fig. 3a). Subsequent analyses were performed using mouse iPSCs (mouse_iPS_1 and mouse_iPS_2) obtained from the GEO database. Principal component analysis (PCA) revealed differences in the gene expression status of each cell group (Supplemental Fig. 3b).

A comparison of the fibroblast and iPSC gene expression profiles revealed the shared upregulation of 631 genes in *M. minutoides* and *M. musculus* iPSCs (Fig. 3c). Table 1 shows some of the upregulated genes that are relevant to pluripotency in *M. minutoides*, *M. musculus*, or both iPSC types. Enrichment analysis of these genes revealed significant associations with GO terms related to pluripotency and the maintenance of stem cell populations, as illustrated in Fig. 3c. Conversely, the expression levels of 582 genes decreased in *M. minutoides* and *M. musculus* iPSCs, and this gene cluster encompassed numerous GO terms associated with fibroblast and connective tissue functions (Fig. 3d).

**Table 1:** Some of the significantly upregulated genes in *M. minutoides* and *M. musculus* iPSCs.

Gene	iPSCs	
	*Mus minutoides*	*Mus musculus*
Naïve marker		
* Zfp42*	○	○
* Dppa3*	○	○
* Tbx3*	○	○
* Dazl*		○
* Pecam1*		○
Primed marker		
* Otx2*	○	○
* Fgf5*	○	
* Foxa2*	○	
* Nodal*		○
* Lefty1*		○
* Lefty2*		○

### Differentiation potential of iPSCs

**Figure 4:**
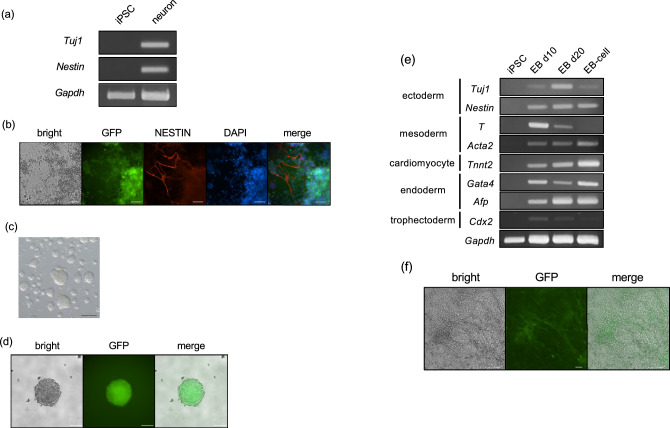
In vitro differentiation potential analysis of *Mus minutoides* induced pluripotent stem cells (iPSCs). (**a**) RT-PCR analysis of neuronal differentiation-induced *M. minutoides* iPSCs. *Gapdh* was used as the positive control. (**b**) Fluorescence immunostaining in neuronal differentiation-induced *M. minutoides* iPSCs with the anti-NESTIN antibody. Bar: 50 µm. (**c**) Bright field image of embryoid bodies (EBs) formed from *M. minutoides* iPSCs on day 4 of floating culture. Bar: 200 µm. (**d**) Fluorescence microscopic image of *M. minutoides* EBs on day 5 of floating culture. Bar: 100 µm. (**e**) RT-PCR analysis of EBs (EB d10, EB d20) and EB migrating cells (EB-cells) cultured for 10 or 20 days. *Gapdh* was used as the positive control. (**f**) Fluorescence microscopic image of *M. minutoides* EB-cells differentiated into cardiomyocytes. Bar: 100 µm.

To assess the differentiation potential of *M. minutoides* iPSCs, we conducted in vitro differentiation experiments targeting the neuronal lineages. Although a considerable number of cells underwent apoptosis during the early stages of differentiation, the expression of neuronal marker genes (*Tuj1*, *Nestin*) was observed 14 days post-induction (Fig. 4a). Fluorescent immunostaining also revealed elongated processes originating from NESTIN-positive neurons (Fig. 4b). EB formation was examined in *M. minutoides* iPSCs. These iPSCs successfully formed EGFP-expressing EBs after 5 days of culture under EB-forming conditions (Fig. 4c,d). Using RT-PCR, we found that the EBs and cells migrating from the EB masses differentiated into three germ layers, the ectoderm, mesoderm, and endoderm, as well as the trophic ectoderm, which contributes to extraembryonic tissues (Fig. 4e).

We also performed an adhesion culture of EBs derived from *M. minutoides* iPSCs. A population of cells with filament-like structures and self-beating behavior, characteristic of cardiomyocytes, was observed within the layered sheets of adherent cells (Fig. 4f and Supplemental Movie 1). Based on RT-PCR using this cell population, *Tnnt2*, a specific marker of cardiomyocytes, was robustly expressed (Fig. 4e).

### Teratoma

**Figure 5:**
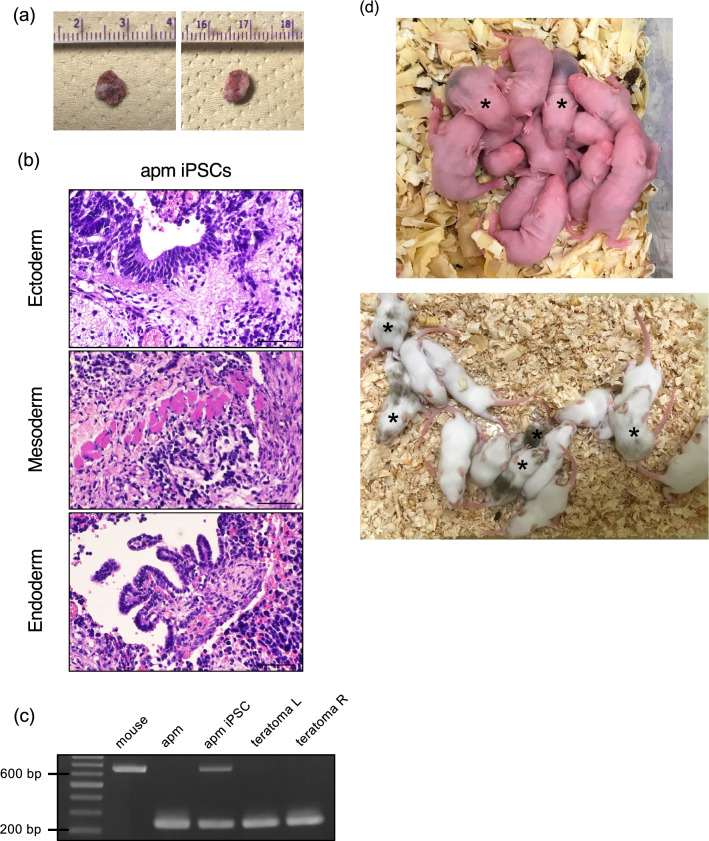
In vivo differentiation and contribution potential analysis of *Mus minutoides* induced pluripotent stem cells (iPSCs)*.* (**a**) Teratoma formed from *M. minutoides* iPSCs. (**b**) Ectoderm (neural epithelium), mesoderm (muscle), and endoderm (gastrointestinal epithelium) observed in the teratoma. Bar: 50 µm. (**c**) Identification of the cell origin based on PCR using genomic DNA. “mouse" and "apm" represent genomic DNA extracted from *M. musculus* and *M. minutoides*, respectively. (**d**) The upper and lower images represent 3- and 11-day-old mice, respectively. The coat color (wild color) from *M. minutoides* is observed with the coat color (white) from ICR (*M. musculus*). Representative chimeric mice are marked with *.

To evaluate the in vivo differentiation potential of *M. minutoides* iPSCs, *M. minutoides* iPSCs were subcutaneously injected into nude mice with specific immune cell dysfunction. Two weeks after the injection, teratoma formation was observed (Fig. 5a). Subsequent histological analysis revealed that these teratomas contained tissues representing all three germ layers: neural tissue (ectoderm), muscle (mesoderm), and intestinal epithelium (endoderm) (Fig. 5b). To determine the genomic origin of the teratomas, we extracted genomic DNA from paraffin-embedded sections to detect *M. minutoides* iPSC colonies. Our analyses confirmed that the genomic DNA of teratomas corresponded to that of *M. minutoides* (Fig. 5c).

### Chimeric mice

To evaluate the developmental contribution of *M. minutoides* iPSCs in animals, we used the blastocyst complementation method to generate chimeric mice. By integrating *M. minutoides* iPSCs into *M. musculus* (ICR mouse) blastocysts, chimeric pups with *M. minutoides* coats were successfully generated and allowed to develop for two weeks (5/14 pups) (Fig. 5d).

## Discussion

In the present study, we successfully generated iPSCs from *M. minutoides* fibroblasts, which formed dome-shaped colonies. In addition, we demonstrated that *M. minutoides* iPSCs are pluripotent and can contribute to the generation of chimeric mice when transplanted into the embryos of *M. musculus*, a closely related species within the *Mus* genus.

To establish *M. minutoides* iPSCs, we used a combination of seven genes, including Yamanaka factors (*Oct3/4*, *Sox2*, Klf4, *c-Myc*), *Glis1*, *Nanog*, and *Lin28a*, which enhance the efficiency of iPSC generation^26,27^. Previously unpublished data revealed successful iPSC generation from *M. musculus* (C57BL/6N, C57BL/6 J, ICR) and *Rattus norvegicus* (Wistar-Imamichi) using the same vector. Although the gene sequences necessary for iPSC establishment in *M. minutoides* are currently unknown, we used vectors containing *Homo sapiens* sequences to facilitate successful conversion of *M. minutoides* fibroblasts into undifferentiated cells. Owing to the close evolutionary relationship between *M. minutoides* and *M. musculus*^9^, the successful establishment of *M. minutoides* iPSCs in this study is a significant achievement. The successful establishment of *M. minutoides* iPSCs was largely due to the use of highly efficient vectors that combine seven factors. The expression of EGFP was observed in colonies and persisted after passaging. PCR was performed to confirm that the iPSC colonies established in this study were derived from *M. minutoides* and not *M. musculus*. The difference in the length of the *Ghr* promoter region between *M. minutoides* and *M. musculus*, as observed in our previous study (unpublished data), was used to identify *M. musculus* and *M. minutoides* DNA via PCR, with the genome as a template. The genomic DNA extracted from *M. minutoides* iPSCs amplified not only *M. minutoides* DNA but also a small amount of *M. musculus*-derived products, which are speculated to be of feeder cell origin. Attempting a feeder-free culture of *M. minutoides* iPSCs was unsuccessful due to a decline in growth rate and a reduction in the number of colonies during cultivation.

We analyzed the gene expression profiles of *M. minutoides* iPSCs and observed the expression of various pluripotency marker genes. Immunostaining revealed the presence of NANOG and OCT3/4 in EGFP-positive *M. minutoides* iPSCs. Moreover, the colonies were observed to maintain their undifferentiated state. Although we did not determine whether the gene expression was endogenous or exogenous, interestingly, *Eras* and *Fgf4*, which are not expressed in the gene expression vectors commonly used for iPSC induction, were expressed. This finding suggests that the induction vectors employed in our study initiated reprogramming in *M. minutoides* fibroblasts. Based on *Fgf4* sequence analysis, although the amino acid sequence remained identical, the cDNA sequence partially differed. This disparity in cDNA sequences indicated that fibroblast initialization triggered the reactivation of genes associated with the original pluripotent state specific to *M. minutoides*. Prior to induction, we observed the expression of the *Klf4* gene in fibroblasts despite its inclusion in the gene expression vectors employed for iPSC induction. The *Klf4* gene was originally isolated from NIH3T3, a cell line derived from mouse fetal skin^28^. Notably, *Klf4* is expressed in the dermal fibroblasts of *M. musculus* and the fibroblasts of *Tokudaia tokunoshimensis* (Amami spiny rat) and *Myotis lucifugus* (little brown bat), which have been successfully used to generate iPSCs using similar induction methods^23,29^. These observations strongly suggest that *Klf4* may also serve as an endogenous gene expressed in *M. minutoides* tail fibroblasts. *Klf4* plays diverse roles, including the maintenance of skin barrier function. In stem cells, *Klf4* actively regulates pluripotency gene expression and inhibits the induction of differentiation-related genes^30^. Therefore, *Klf4* may exhibit different functions between *M. minutoides* iPSCs and fibroblasts.

Quantitative analysis revealed differences in the expression of pluripotency markers between the *M. minutoides* iPSC lines. In particular, the expression levels of the pluripotency marker genes, particularly *Nanog*, were nearly identical between the two iPSC lines (apm_iPSC_6 and iPSC_10) and *M. musculus* ES cells. In these two iPSC lines, the expression levels of *Oct3/4*, *Sox2*, *Eras*, and *Fgf4* were significantly higher than those in *M. musculus* ESCs. Notably, the expression levels of *Fgf4* were substantially high. *Fgf4* is involved in the proliferation and differentiation of ESCs and tissue stem cells in mice^31^. Moreover, the absence of *Fgf4* impairs differentiation into nerve and mesoderm lineages^32^, indicating its importance in specific differentiation. Based on our findings, the *M. minutoides* iPSCs established in this study readily differentiated into neurons, and a substantial number of cardiomyocyte colonies emerged from migrating cells within the EBs. This enhanced differentiation potential may be attributed to the elevated expression of *Fgf4*. RNA-seq analysis also revealed significant differences in gene expression between the established *M. minutoides* iPSCs and fibroblasts, highlighting the expression of numerous pluripotency-related genes that could not be elucidated by RT-PCR. Principal component analysis using gene expression data from *M. musculus* iPSCs (mouse_iPS_1, mouse_iPS_2) and fibroblasts (mouse_fib_1, mouse_fib_2) revealed that *M. minutoides* iPSCs and *M. musculus* iPSCs have different gene expression states. Moreover, distinct gene expression patterns were discovered between the fibroblasts of *M. minutoides* and *M. musculus*. Although we cannot definitively exclude the possibility that these differences are due to technical variations in the extraction processes, the availability of *M. musculus* iPSCs data from the GEO database implies that each cell population of *M. minutoides* and *M. musculus* may possess a unique gene expression profile that must be further investigated. In the enrichment analysis, the highest-ranked pathway matched pluripotency, and many other GO terms related to embryonic development and the maintenance of undifferentiated potential were observed. Based on the RT-qPCR results, a strong positive correlation was found among the expression levels of the pluripotency markers, suggesting that the regulatory mechanism governing pluripotency gene expression in *M. minutoides* was similar to that observed in *M. musculus*^33,34^; however, of the 292 genes included in the top hit WP1763: Mechanisms associated with pluripotency, only 102 genes (104 genes in iPSC_6 and 109 in iPSC_10) had significantly increased expression in *M. minutoides* iPSCs. As shown in Fig. 3c,d, 1661 upregulated and 1227 downregulated genes were unique to *M. minutoides* iPSCs. Thus, although *M. minutoides* belongs to the same genus (*Mus*) as mice, pathways involved in maintaining pluripotency in *M. minutoides* may differ from those in mice. GO:0006305 DNA alkylation is attributed to mitomycin C-treated feeder cells.

We proceeded to evaluate the pluripotency of *M. minutoides* iPSCs. Based on the in vitro results, these iPSCs successfully differentiated into neurons, exhibiting numerous elongated processes originating from dome-shaped colonies. This successful neural differentiation suggests that functional cells can be directly derived from the *M. minutoides* iPSCs established in this study. Subsequently, the floating culture method was employed to generate EBs from *M. minutoides* iPSCs. EBs are three-dimensional cellular aggregates formed from floating PSC cultures. EBs possess a two-layered structure comprising the EB ectoderm, which differentiates into neurons and other cell types, and the primitive endoderm, which differentiates into the mesoderm and endoderm. Using a floating-culture technique, we successfully formed EBs from *M. minutoides* iPSCs. These EBs exhibited a cell mass, with initiation of the cellular fate of the three germ layers (ectoderm, mesoderm, and endoderm). These results indicate that *M. minutoides* iPSCs are pluripotent and can differentiate into diverse cell lineages in vitro. Although EBs primarily differentiate into cell lineages comprising the EB, our gene expression analysis revealed slight expression of *Cdx2*, a marker associated with the trophic ectoderm. Although further detailed analysis of this finding is warranted, it highlights an intriguing outcome regarding EB differentiation in *M. minutoides*. The expression of the *T* (*Brachyury*) gene decreased significantly as the EB culture period progressed. *T* is a transcription factor expressed during the early stages of mesoderm formation^35^. According to previous reports, the expression of *T* is initially upregulated during the early culture of EBs derived from mouse ESCs, followed by a gradual decrease^36^. In EBs derived from *M. minutoides* iPSCs, an extended culture period resulted in the differentiation of EBs accompanied by a decrease in *T* expression, indicating differentiation into the mesoendodermal lineage, similar to *M. musculus* iPSCs.

In the present study, *M. minutoides* iPSCs differentiated into three germ layers in vivo, similar to *M. musculus* ESCs. Chimeric mice were successfully generated by injecting *M. minutoides* iPSCs into *M. musculus* blastocysts. The resulting chimeras exhibited a predominantly white coat color, characteristic of ICR (*M. musculus*), with additional patches displaying a wild coat color derived from *M. minutoides*. This observation indicates that *M. minutoides* iPSCs actively contribute to inner cell mass formation in conjunction with *M. musculus*-derived cells, demonstrating their functional and physiological capabilities in vivo.

PSCs are classified into two distinct states, naïve and primed, based on their morphological characteristics and capacity to form chimeric organisms^37^. For *M. musculus*, ESCs are recognized as naïve whereas epiblast stem cells (EpiSCs) are considered primed. These two states exhibit notable differences in their genetic expression profiles and their ability to contribute to chimerism^38,39^. RNA-seq analysis revealed that certain genes that were highly expressed in *M. musculus* ESCs, such as *Zfp42*, *Dppa2*, and *Tbx3*, were also significantly upregulated in *M. minutoides* iPSCs, whereas other genes were not significantly upregulated. Conversely, among the genes highly expressed in *M. musculus* EpiSCs, *Fgf5* and *Foxa2* exhibited significantly higher expression in *M. minutoides* iPSCs, whereas no significant differences were observed in *M. musculus* iPSCs. Considering the colony morphology and outcomes obtained in our study, including the ability to contribute to chimeric mice, the established *M. minutoides* iPSCs can be inferred to possess properties that are either comparable to or very closely aligned with those of naïve PSCs.

In summary, the findings of our in vitro and in vivo investigations confirmed that *M. minutoides* iPSCs generated by reprogramming *M. minutoides* fibroblasts possess the essential characteristics of PSCs and contribute to their ontogeny. The establishment of *M. minutoides* iPSCs has remarkable potential to advance the analysis of dwarfism, which is the most distinct *M. minutoides* phenotype. Thus, the biology of non-model animals that lack established developmental engineering techniques and experience challenges in terms of ESC establishment can be explored, which may also advance research on interspecies chimera creation.

## Methods

### Animal experiment

*Mus minutoides* (obtained from the Pet Shop YANOHASHI) was exsanguinated via cervical dislocation under anesthesia with medetomidine, midazolam, and butorphanol and used for each experimental procedure. The animal experiments complied with the regulations and guidelines of the Yamaguchi University Experimental Animal Committee (No. 291) and the Animal Care and Use Committee of the University of Tokyo (P22-092), and their protocols were duly approved by the respective committees. The results of the study were also reported in accordance with the ARRIVE guidelines.

### Fibroblast establishment

Primary fibroblasts were isolated from the tail tips of dead *M. minutoides* and *M. musculus*. The tail tip was attached to gelatin-coated plates and cultured in Dulbecco’s modified Eagle’s medium (DMEM; FUJILIM Wako) supplemented with 10% fetal bovine serum (FBS), penicillin (100 U/mL), and streptomycin (100 µg/mL) at 37 °C with 5% CO_2_. The culture medium was replaced weekly, and the cells were passaged as necessary to monitor their migration.

### Plasmid construction

PiggyBac inverted terminal repeat (ITR) sequences were synthesized by overlap extension PCR using oligonucleotides obtained from Eurofins (Japan). The amplicons were inserted into the AatII–AflIII sites of pUC19 to construct pUC19-PB. To construct the plasmid, PB-TRE-Oct3/4-2A-Klf4-2A-Sox2-2A-cMyc (PB-TRE-OKSM), we cloned the TRE-CMV minimal promoter and a polyA signal sequence fragment from a previously constructed plasmid DNA^40^. The OKSM fragment was obtained from pMaster3 (Addgene plasmid #58526)^41^, which was modified from a previously reported plasmid^42^. These fragments were inserted into the ITRs of pUC19-PB.

Similarly, to construct PB-TRE-Glis1-2A-Nanog-2A-lin28a (PB-TRE-GNL), the TRE-CMV minimal promoter and poly (A) signal sequence fragments were obtained as described above. The *Glis1* open reading frame (ORF) was cloned using C57BL/6-derived testis cDNA, and the NL fragment was obtained from pMaster3 (Addgene plasmid #58526)^41^. These fragments were inserted into the ITRs of pUC19-PB.

To construct PB-CAG-rtTA-IRES-eGFP-pGK-NeoR, the CAG-rtTA and IRES-eGFP sequences were obtained from a previously constructed plasmid DNA^40^. These fragments, along with pGK-NeoR (Addgene plasmid #13442)^43^, were inserted into pUC19-PB.

The above plasmids will be deposited in Addgene.

For pCAG-T3-hyPBase-pA construction, the hyPBase ORF was cloned from pCMV-hyPBase (kindly provided by Dr. A Bradley, Wellcome Sanger Institute) using PCR and inserted into the NotI–ClaI site of pCAG-T3-hCas9-pA (Addgene plasmid #48625)^44^.

The constructed vectors were sequenced using a commercial sequencing kit (Applied Biosystems, Foster City, CA, USA) and a DNA sequencer (Applied Biosystems), according to the manufacturer’s instructions. All iPSC-inducing plasmid constructs are shown in Fig. 1a.

### iPSC establishment

Fibroblasts from *M. minutoides* were dissociated, washed, and suspended in Opti-MEM (Gibco). The cell suspension was then transferred to a 4-mm cuvette electrode (SE-204; BEX Co., LTD), and 2.5 μg/100 μL of plasmid was added individually for iPSC induction (PB-TRE-OKSM, PB-TRE-GNL, PB2-CAG-rtTA-ires-eGFP pGK-Neo, and pCAG-T3-hyPBase-pA(95)). Electroporation was performed using a CUY21EDIT II electroporator (BEX) with a single 10 ms pulse at 350 V, followed by five 50 ms pulses at 40 V at 50 ms intervals. After electroporation, the cells were reseeded on feeder cells in ESGRO Complete Basal Medium (Merck) supplemented with penicillin (100 U/mL), streptomycin (100 μg/mL), 20% KnockOut Serum Replacement (KSR) (Gibco), and ESGRO-2i Supplement Kit (Merck), with 2 μg/mL (final concentration) doxycycline (DOX) (Takara). Emerging colonies with iPSC-like morphology were passaged on feeder cells and mitomycin C-treated mouse embryonic fibroblasts and cultured with DOX until the reappearance of the colonies. Subsequently, DOX supplementation was discontinued, and the cells were further cultured. Individual colonies that maintained domed-morphology were selected under a stereomicroscope and single cells from a colony were seeded on feeder cells in 96-well plates to establish multiple *M. minutoides* iPSC lines.

### Genomic PCR

Primers were designed based on the promoter region sequence of the *M. musculus growth hormone receptor* (*Ghr*) gene, and genomic PCR was performed using BIOTAQ DNA polymerase (NIPPON Genetics). The primer sequences are listed in Table 2. The following PCR cycling conditions were employed: initial denaturation at 94 °C for 3 min, followed by 35 cycles of denaturation at 94 °C for 30 s, annealing at 64 °C for 45 s, extension at 72 °C for 45 s, and a final extension at 72 °C for 1 min.

**Table 2:** Primer sequences for PCR conducted with genomic DNA.

Gene	Primer sequences (5ʹ → 3ʹ)
*Ghr* pmr	
F	TCCCTGCAGACCTGTGTTCCGTAC
R	GCACCTCCTGCAGGTACAGGAAG

### Alkaline phosphatase staining

Alkaline phosphatase (ALP) staining was conducted using an Alkaline Phosphatase detection kit (Merck), according to the manufacturer’s protocol.

### Genomic DNA and mRNA extraction and reverse transcription

Total RNA and genomic DNA (gDNA) were extracted using the NucleoSpin TriPrep kit (Takara) or ReliaPrep RNA Cell Miniprep System (Promega), respectively, according to the respective protocols. Total RNA was reverse-transcribed using the QuantiTect Reverse Transcription Kit (QIAGEN), according to the manufacturer’s protocol.

### RT-PCR

The primer design for RT-PCR was based on highly conserved regions identified by comparing the DNA sequences of *M. musculus*, *Mus pahari*, and *Mus spretus*. Primers were designed for the pluripotency markers: *Nanog*, *Oct3/4*, *Sox2*, *Klf4*, *Eras*, and *Fgf4*; neural markers: *Tuj1* and *Nestin*; markers for ectoderm (*Tuj1*, *Nestin*), mesoderm (*T*, Acta2), cardiomyocyte (Tnnt2), endoderm (*Gata4*, *AFP*), and trophectoderm (*Cdx2*) differentiation in embryoid bodies (EBs) and migrating cells; and primers for the *Gapdh* gene. The primer sequences are listed in Table 3. PCR amplification was performed using BIOTAQ DNA polymerase (Nippon Genetics) and the following cycling conditions: initial denaturation at 94 °C for 3 min, followed by 35 cycles of denaturation at 94 °C for 30 s, annealing at 64 °C for 45 s, extension at 72 °C for 45 s, and a final extension step at 72 °C for 1 min.

**Table 3:** Primer sequences for RT-PCR.

Gene	Primer sequences (5' → 3')
*Nanog*	
F	GATGCGGACTGTGTTCTCTCAGG
R	TCCAAATTCACCTCCAAATCACTGGC
*Oct3/4*	
F	GCTAGAACAGTTTGCCAAGCTGC
R	TGCACCAGGGTCTCCGATTTGC
*Sox2*	
F	GGATAAGTACACGCTTCCCGGAG
R	TAGGACATGCTGTAGGTGGGCG
*Klf4*	
F	GCGAGTCTGACATGGCTGTCAG
R	GTTGTTACTGCTGCAAGCTGCAC
*Eras*	
F	GCTCTCACCATCCAGATGACTCAC
R	CTCTGAATCTCATGGACAAGCAGG
*Fgf4*	
F	AACGTGGGCATCGGATTCCACC
R	CGTAGGCGTTGTAGTTGTTGGGC
*Gapdh*	
F	GTGCTGAGTATGTCGTGGAGTC
R	CATACTTGGCAGGTTTCTCCAG
*Tuj1*	
F	GGACACCTATTCAGGCCCGACAAC
R	CACGCTGAAGGTGTTCATGATGC
*Nestin*	
F	CGGGAGAGTCGCTTAGAGGTG
R	ATCTTGAGGTGTGCCAGTTGCTG
*T*	
F	CATGCTGCCTGTGAGTCATAACG
R	CCTAGAAGATCCAGTTGACACCGG
*Acta2*	
F	AGCGTGAGATTGTCCGTGACATC
R	CCTTCTGCATCCTGTCAGCAATG
*Tnnt2*	
F	AAGGCTCTGTCCAACATGATGCAC
R	TGATCCGGTTTCGCAGAACGTTG
*Gata4*	
F	GGTAACTCCAGCAATGCCACTAGC
R	CCTGGAAAGGTGTTTGAACAACCCG
*Afp*	
F	AAGCTGCGCTCTCTACCAGAC
R	TGGCACAGATCCTTGTGGAAGATG
*Cdx2*	
F	CTGGAGCTGGAGAAGGAGTTTCAC
R	GCAAGGAGGTCACAGGACTCAAG

### Sequencing

After PCR, bands corresponding to the desired sizes were excised from the electrophoresed gel, and the amplified DNA fragments were extracted using the FastGene Gel/PCR Extraction Kit (Nippon Genetics). The extracted DNA was sequenced at Yamaguchi University Center for Gene Research. The obtained sequence data were aligned at the nucleotide and amino acid levels using CLUSTALW 2.1 software (https://www.genome.jp/tools-bin/clustalw) to determine the corresponding amino acid sequences.

### RT-qPCR

Real-time PCR was conducted using the KAPA SYBR Fast qPCR Kit (Nippon Genetics) with synthesized cDNA as the template. The reaction was performed on the CFX96 Touch Deep Well real-time PCR analysis system (Bio-Rad) using the following cycling conditions: initial denaturation at 95 °C for 3 min, followed by 40 cycles of denaturation at 95 °C for 3 s, and annealing/extension at 60 °C for 20 s. Each gene expression level was normalized to that of *Gapdh* and analyzed using relative cycle threshold (CT). The values are presented as mean ± standard deviation.

### Cellular immunostaining

The cells were fixed with 4% paraformaldehyde for 30 min. After three washes with Tris-buffered saline (TBS), the cells were permeabilized with 0.2% Triton X-100 in TBS (TBST) for 20 min. After three additional washes with TBS, the cells were blocked with 5% goat serum in TBST for 30 min. Rabbit anti-mouse NANOG (diluted 200-fold, ab80892; Abcam) and rabbit anti-human mouse OCT4 (diluted 200-fold, C30A3C1; Cell Signaling Technology) were used as the primary antibodies for the pluripotency markers. In this study, a specific OCT4 antibody was used to recognize the pluripotency-associated OCT4A isoform rather than the more prevalent OCT4B isoform. These primary antibodies were diluted in 5% goat serum in TBST and incubated with the cells for 17 h at 4 °C. Thereafter, Alexa Fluor 594-conjugated goat anti-rabbit IgG (diluted 500-fold, ab150080; Abcam) was used as the secondary antibody. For neuronal differentiation, rat anti-mouse NESTIN antibody (diluted 200-fold, 012-26843, FUJIFILM Wako) was used as the primary antibody, and Alexa Fluor 568-conjugated goat anti-rat IgG (diluted 500-fold, A-11077; Thermo Fisher Scientific) was used as the secondary antibody. Following three additional washes with TBS, contrast staining was performed by incubating the cells with 1 g/mL DAPI in TBS for 15 min under shielded light.

### RNA-seq analysis

Total RNA was extracted from *M. minutoides* iPSCs and fibroblasts using the Maxwell RSC RNA Cell Kit (Promega), and RNA-seq analysis was performed using the NovaSeq 6000 (Illumina) at the Yamaguchi University Center for Gene Research. The number of reads was 33.5 million for fib_1, 33.1 million for fib_2, 36.7 million for iPS-6, and 33.9 million for iPS-10. The expression levels were analyzed after TPM normalization. The RNA-seq data for mouse iPSCs and fibroblasts (GSE46104; GSM1123730, GSM1123731, GSM1123732, and GSM1123733) were obtained from NCBI Gene Expression Omnibus (GEO) database using GEO RNA-seq Experiments Interactive Navigator (GREIN) (http://www.ilincs.org/apps/grein/?gse =).

### Neural differentiation

To induce the differentiation of *M. minutoides* iPSCs into neurons, the culture medium was replaced with the Ndiff227 medium (Takara).

### Embryoid body formation

*Mus minutoides* iPSCs cultured on the feeder cells were detached and collected. The cells were then suspended in DMEM supplemented with 10% KSR, MEM non-essential amino acid solution (100×) (Fujifilm Wako), StemSureR 10 mmol/L 2-mercaptoethanol solution (100×) (Fujifilm Wako), penicillin (100 U/mL), and streptomycin (100 µg/mL). The supernatant containing floating iPSCs was collected and cultured in a suspension for EB formation. These EBs were subjected to adherent culture to obtain migrating cells. Total RNA was extracted from EBs, and migrating cells were derived from EBs and reverse-transcribed.

### Teratoma formation

A total of 2 × 10^5^ cells were collected, suspended in Matrigel (Corning), and subcutaneously injected into 8-week-old KSN male mice. After a 2-week incubation period, the teratomas were extracted, fixed with 3.7% paraformaldehyde, embedded in paraffin, sliced into 4 µm-thick sections, and stained with hematoxylin and eosin. Genomic DNA was extracted from teratomas using the NucleoSpin DNA FFPE XS kit (Takara), according to the manufacturer’s protocol.

### Chimeric mice production

Interspecies chimeras between *M. musculus* and *M. minutoides* were generated using blastocyst complementation, according to the protocols described in Nagy et al.^45^, with some modifications. Briefly, ICR zygotes were obtained via in vitro fertilization^46^ and cultured in KSOMaa-BSA medium until the blastocyst stage. *M. minutoides* iPSCs were microinjected into the blastocoel of 4.0–4.5 dpc ICR blastocysts, which were then transferred into the uteri of pseudopregnant recipient ICR females on day 2.5 of the pseudopregnancy, the pups were obtained through natural birth.

### Statistical analysis

Statistical analysis was performed using a *t*-test to determine significant differences between values. *p* < 0.05 was considered statistically significant.

### Supplementary Information

Supplementary Information 1.

Supplementary Information 2.

Supplementary Movie 1.
