# Supplementary material for: Establishment of African pygmy mouse induced pluripotent stem cells using defined doxycycline inducible transcription factors
Source: Sci Rep. 2024 Feb 8;14:3204. doi: 10.1038/s41598-024-53687-9 (PMC10853177; doi:10.1038/s41598-024-53687-9)

FIGURE 1 Establishment of *M. minutoides* iPSCs

(f)

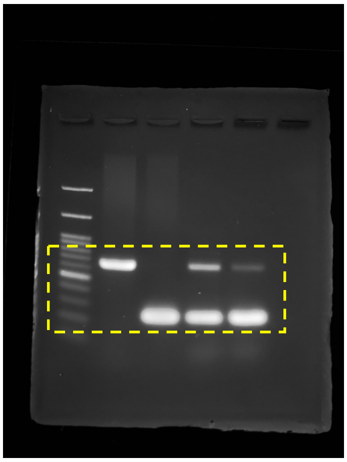

FIGURE 2 Analysis of pluripotency in *M. minutoides* iPS cells

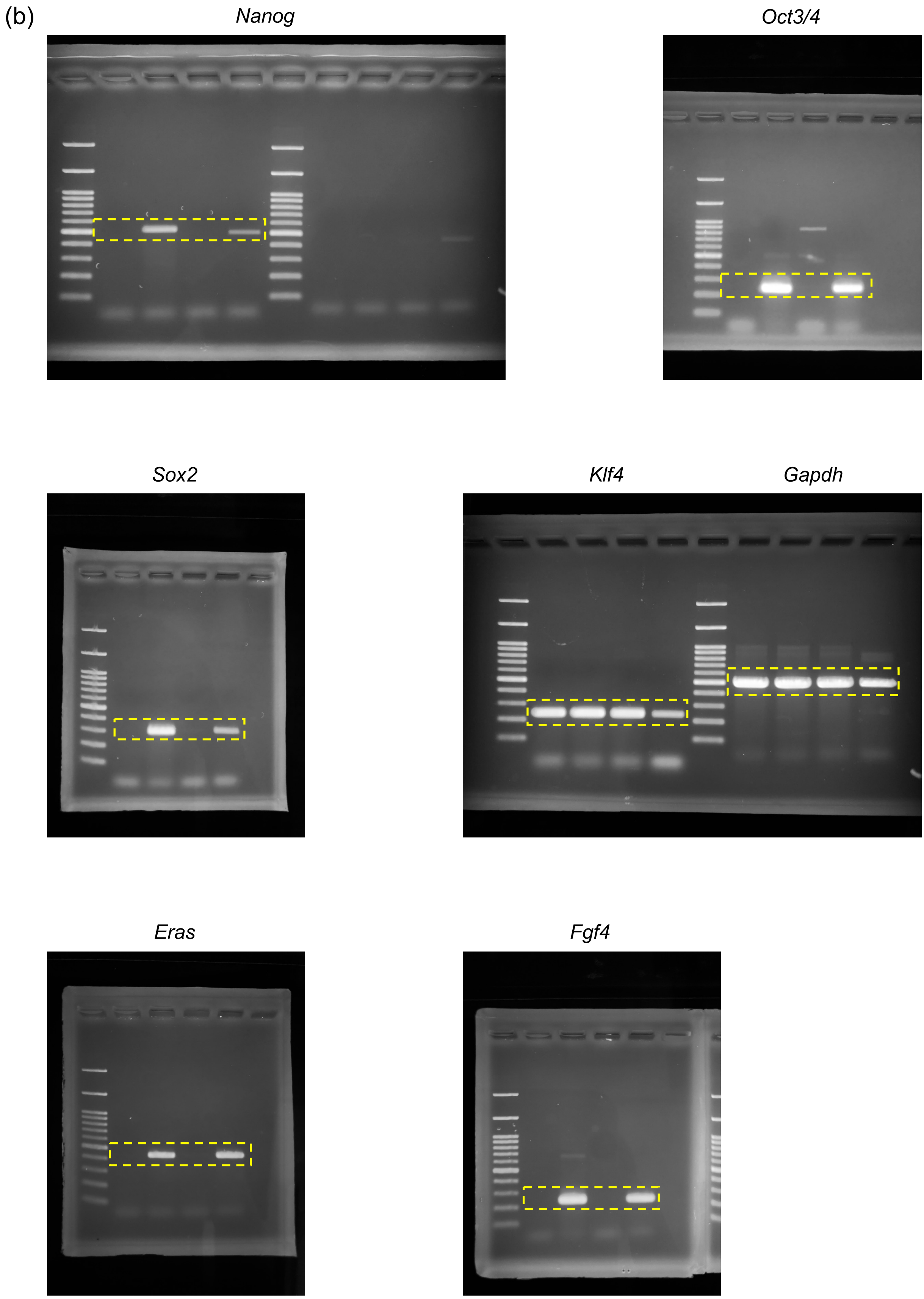

FIGURE 4 Differentiation potential analysis of *M. minutoides* iPS cells *in vitro*

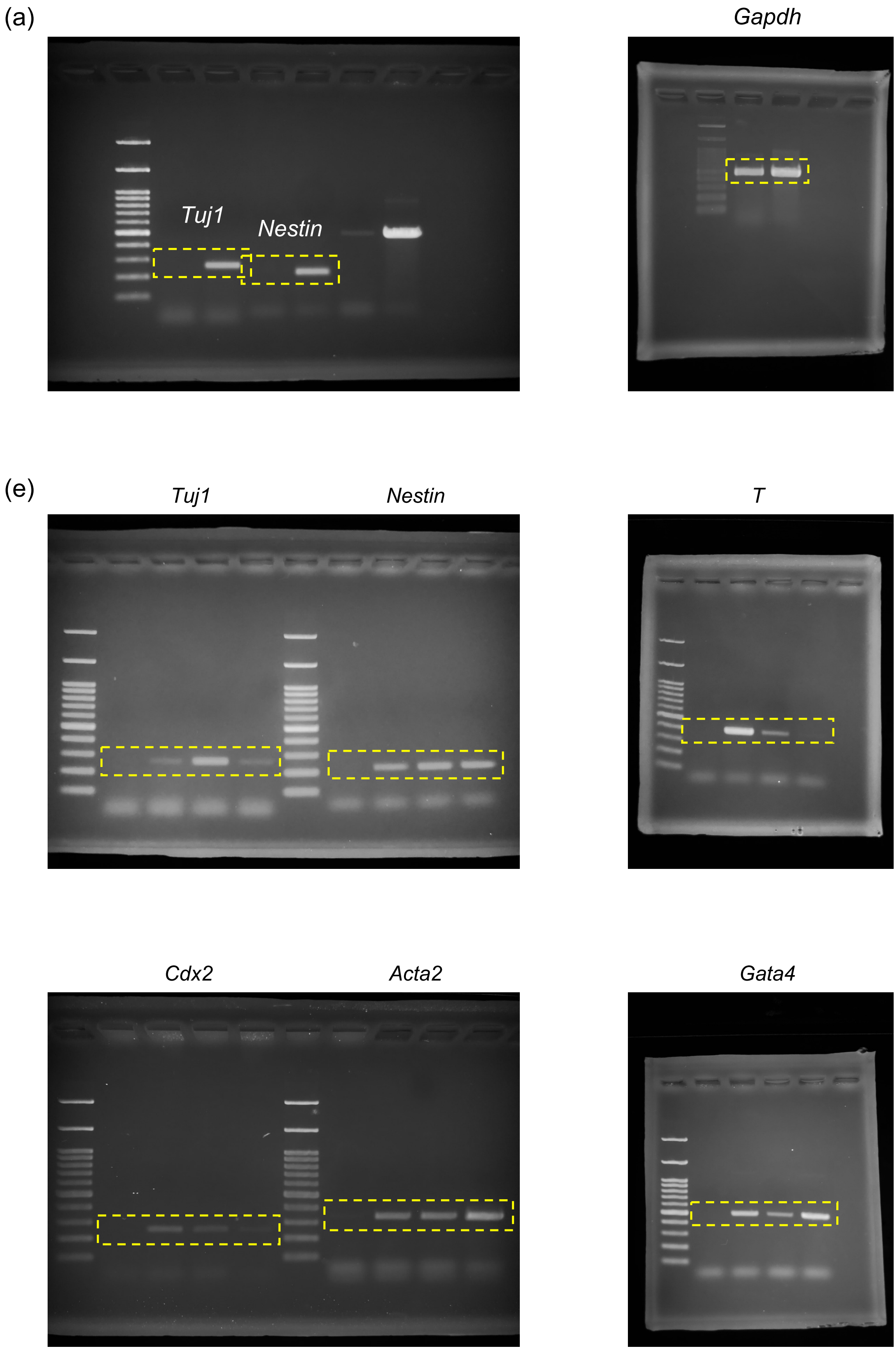

FIGURE 4 Differentiation potential analysis of *M. minutoides* iPS cells *in vitro*

(e)

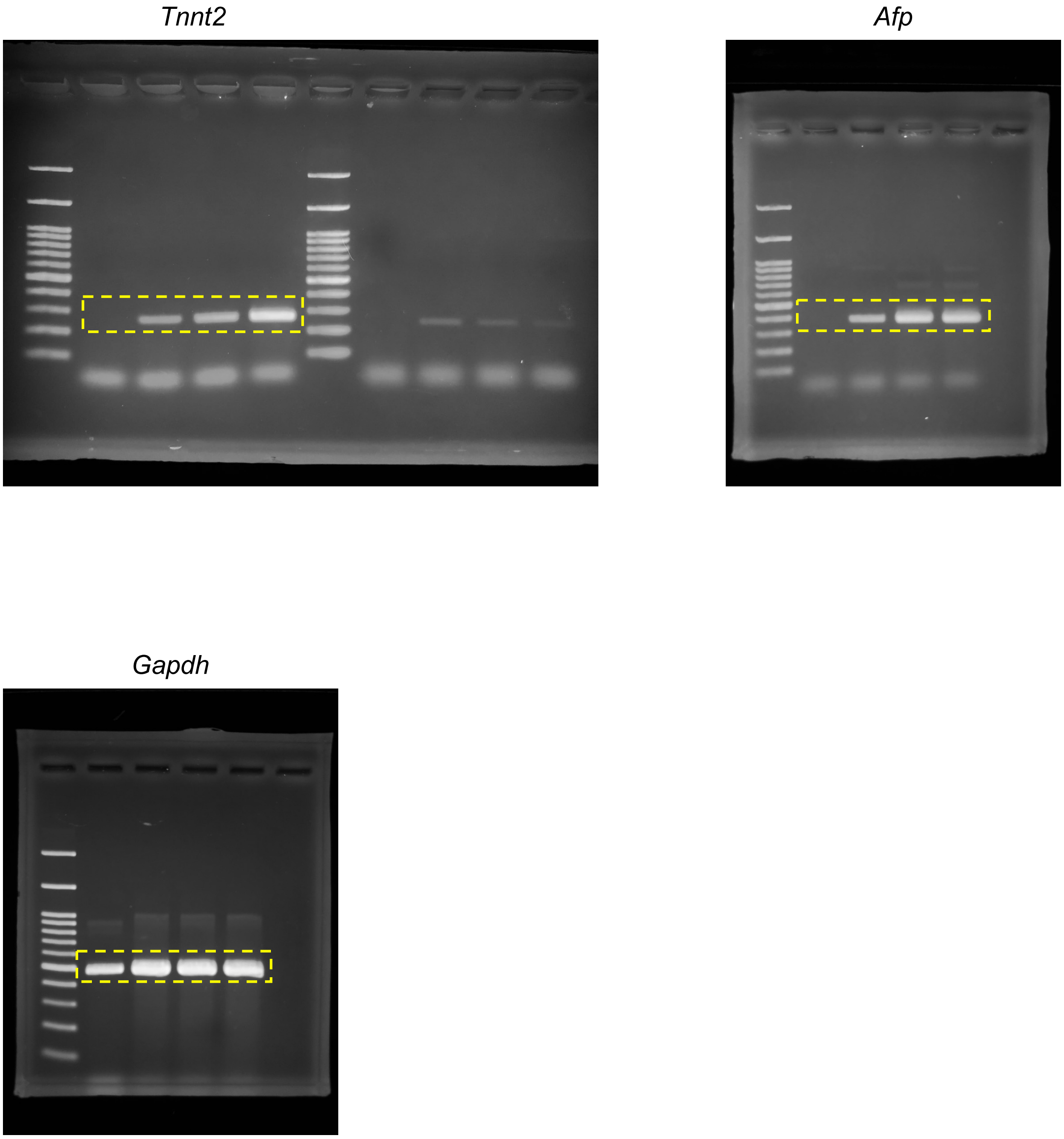

FIGURE 5 Differentiation and contribution potential analysis of *M. minutoides* iPS cells *in vivo*.

(c)

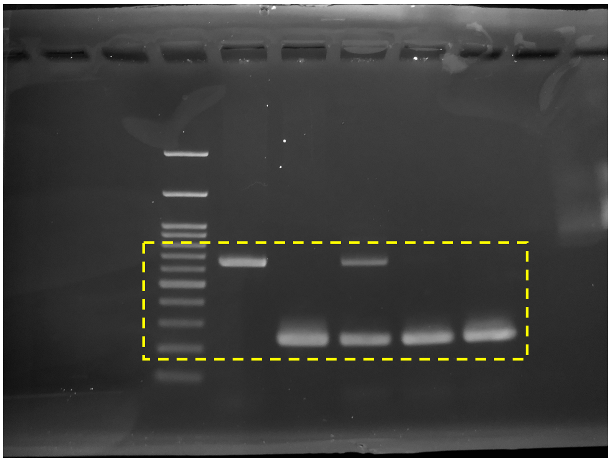

Supplement: Supplementary file 1 — Supplementary Information 1. [file 41598_2024_53687_MOESM1_ESM.pdf]
