# Supplementary material for: Establishment of African pygmy mouse induced pluripotent stem cells using defined doxycycline inducible transcription factors
Source: Sci Rep. 2024 Feb 8;14:3204. doi: 10.1038/s41598-024-53687-9 (PMC10853177; doi:10.1038/s41598-024-53687-9)
Supplement: Supplementary file 2 — Supplementary Information 2. [file 41598_2024_53687_MOESM2_ESM.pdf]

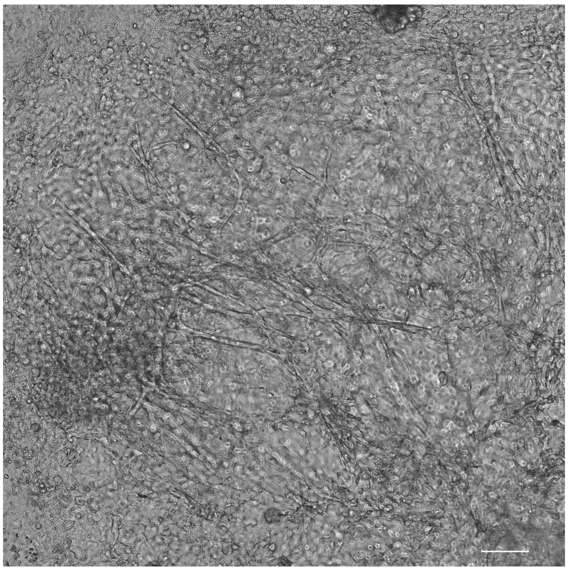

SUPPLEMENTAL MOVIE 1 Cardiac differentiation of migrating cells from *Mus minutoides* embryoid bodies (EBs).

(a)

```
Mus_musculus        TGCAGGTGCTGCCCGACGGCCGCATCGGTGGTGTGCACGCAGACACGAGGGACAGTCTTC
Mus_minutoides      TGCAGGTGCTGCCCGACGGCCGCATCGGTGGTGTGCACGCGGACACAAGGGACAGTCTTT
                    **************************************** ***** ************ 

Mus_musculus        TGGAGCTCTCTCCGGTGCAGCGAGGCGTGGTGAGCATCTTCGGAGTGGCCAGCCGGTTCT
Mus_minutoides      TGGAGCTCTCTCCGGTGCAGCGAGGCGTGGTGAGCATCTTCGGAGTGGCCAGTCGGTTCT
                    **************************************************** *******

Mus_musculus        TCGTGGCCATGAGCAGCAGGGGCAAGCTCTTCGGTGTGCCTTTCTTTACCGACGAGTGTA
Mus_minutoides      TCGTGGCCATGAGCAGCAGGGGCAAGCTCTTCGGTGTGCCTTTCTTTACCGACGAGTGTA
                    ************************************************************

Mus_musculus        AATTCAAAGAAATACTTCT
Mus_minutoides      AATTCAAGGAAATACTTCT
                    ******* ***********
```

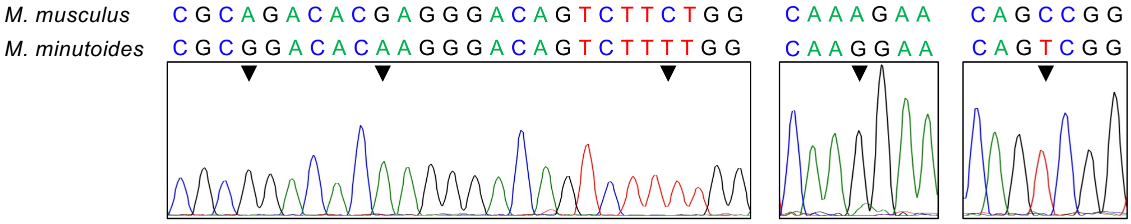

(b)

```
Mus_musculus        VGIGFHLQVLPDGRIGGVHADTRDSLLELSPVQRGVVSIFGVASRFFVAMSSRGKLFGVP
Mus_minutoides      VGIGFHLQVLPDGRIGGVHADTRDSLLELSPVQRGVVSIFGVASRFFVAMSSRGKLFGVP
                    ************************************************************

Mus_musculus        FFTDECKFKEILLPNNYNAY
Mus_minutoides      FFTDECKFKEILLPNNYNAY
                    ********************
```

SUPPLEMENTAL FIGURE 1 Sequence of the *Fgf4* gene in *Mus minutoides*. (a) Alignment of the *Fgf4* cDNA sequences from *M. minutoides* and *M. musculus*. (b) Alignment of the predicted FGF4 amino acid sequences in *M. minutoides* and *M. musculus*. *M. musculus* and *M. minutoides* sequences are shown in the upper and lower rows, respectively. * indicates a sequence match; areas where sequences differ are indicated in red.

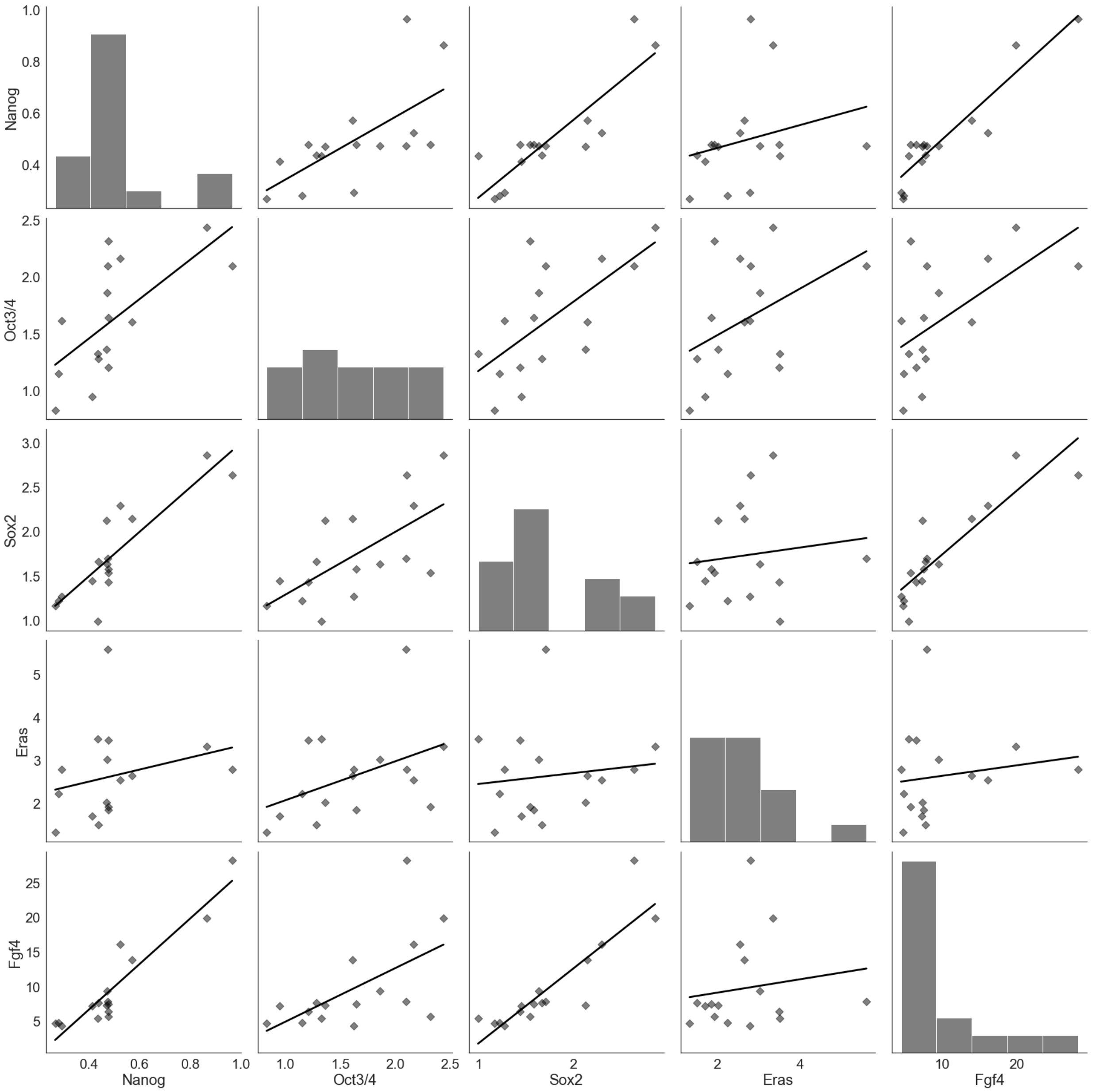

SUPPLEMENTAL FIGURE 2 Scatter plot matrix of pluripotency marker expression levels in *Mus minutoides* induced pluripotent stem cells (iPSCs). Scatter plots of the expression levels of each pluripotency marker per cell line in *M. minutoides* iPSCs and histograms illustrating the frequency of expression levels.

(a)

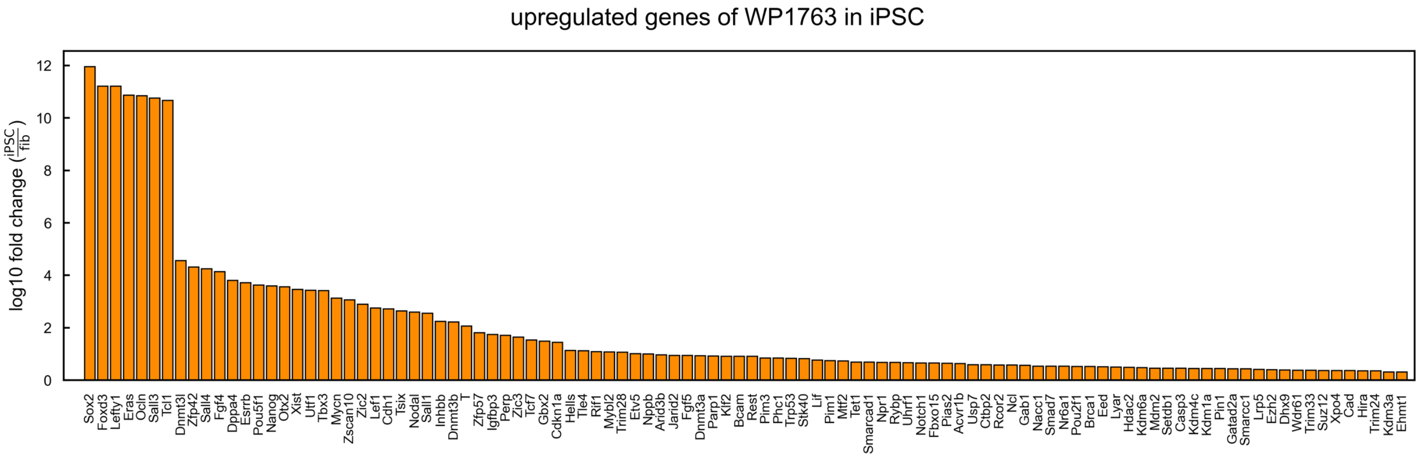

(b)

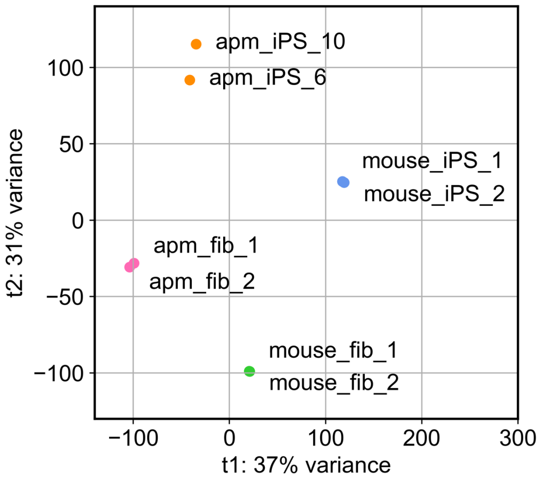

SUPPLEMENTAL FIGURE 3 Supplemental RNA-seq analysis data of *Mus minutoides* induced pluripotent stem cells (iPSCs) and fibroblasts.
(a) Bar plot of the relative expression of the 102 2-fold upregulated genes in *M. minutoides* iPSCs included in WP1763.
(b) Principal component analysis (PCA) of global gene expression in *M. minutoides* fibroblasts (apm_fib_1, apm_fib_2), iPSCs (apm_iPSC_6, apm_iPSC_10), *M. musculus* fibroblasts (mouse_fib_1, mouse_fib_2), and iPSCs (mouse_iPS_1, mouse_iPS_2).
